# Record linkage in public health datasets: a practical experience in a fast in-process analytical database

**DOI:** 10.1590/1980-549720250053

**Published:** 2025-11-28

**Authors:** Wagner Tassinari, Caroline Dias Ferreira, Eugênio Araújo, Débora Medeiros de Oliveira e Cruz, Gislani Mateus Oliveira Aguilar, Oswaldo Gonçalves Cruz, Valéria Saraceni, Antônio Pacheco

**Affiliations:** 1 Universidade Federal Rural do Rio de Janeiro, Departamento de Matemática – Seropédica (RJ), Brazil.; 2 Fundação Oswaldo Cruz, Instituto Nacional de Infectologia – Rio de Janeiro (RJ), Brazil.; III Secretaria Municipal de Saúde do Rio de Janeiro – Rio de Janeiro (RJ), Brazil.; IV Fundação Oswaldo Cruz, Programa de Computação Científica – Rio de Janeiro (RJ), Brazil.

**Keywords:** Information storage and retrieval, Health information systems, Public health surveillance, Algorithms, Armazenamento e recuperação da informação, Sistema de informação em saúde, Vigilância em saúde pública, Algoritmos

## Abstract

**Objective:**

This study presents the accuracy of an algorithm with a mixed approach for linking the Mortality Information System (SIM) and the Influenza Epidemiological Surveillance Information System (SIVEP-Gripe) records, implemented in DuckDB.

**Methods:**

The proposed algorithm was compared with a previously validated algorithm, in different prevalence scenarios. We employed a hybrid deterministic-probabilistic approach, using similarity metrics such as Jaro and Jaro-Winkler. The study highlights important advantages, including superior processing speed and scalability, maintaining high values in terms of sensitivity, specificity and predictive values.

**Results:**

The DuckDB-based solution processed datasets significantly faster, with execution times up to one hundred times shorter, making it particularly suitable for large-scale, real-time applications.

**Conclusions:**

This study underscores the potential of DuckDB as a high-performance analytical database for efficiently managing complex data integration tasks and highlights its suitability for resource-limited environments in public health, where timely and accurate record linkage is often essential.

## RESUMO

**Objetivo:**

Este estudo apresenta a avaliação de um algoritmo com abordagem mista para a vinculação de registros do Sistema de Informações sobre Mortalidade (SIM) e do Sistema de Informação da Vigilância Epidemiológica da Gripe (SIVEP-Gripe), implementado no banco de dados analítico DuckDB.

**Métodos:**

O algoritmo proposto, que combina técnicas determinísticas e probabilísticas com o uso de métricas de similaridade como Jaro e Jaro-Winkler, foi comparado a uma abordagem previamente validada, considerando diferentes cenários de prevalência.

**Resultados:**

Os resultados demonstram vantagens significativas em termos de desempenho computacional e escalabilidade, mantendo elevados níveis de sensibilidade, especificidade e valores preditivos. A solução baseada no DuckDB processou os conjuntos de dados com tempos de execução até cem vezes menores, mostrando-se especialmente adequada para aplicações em larga escala e em tempo real.

**Conclusões:**

Este trabalho evidencia o potencial do DuckDB como uma alternativa de alto desempenho para o gerenciamento eficiente de tarefas complexas de integração de dados, sendo particularmente vantajoso em ambientes com infraestrutura limitada, realidade comum em contextos da saúde pública, onde a vinculação precisa e oportuna de registros é essencial.

## INTRODUCTION

Record linkage (RL) is an important process in public health as it allows the integration of data from various sources, enhancing data accuracy and enabling more robust analyses^1^. By combining multiple datasets, RL significantly increases the value of existing information by supplying missing data or correcting errors in existing data, generating important covariates, and by using family information to control for unmeasured variables and expand research opportunities^2^. This process is crucial for monitoring diseases, evaluating interventions, and developing evidence-based health policies, as emphasized by Roos et al.^3^.

In the context of the Brazilian Unified Health System (SUS), integrating records to identify those that refer to the same individual or event is essential. A common approach to this integration is deterministic linkage, which uses existing primary keys (unique identifiers) in the databases, such as the Brazilian ID number (CPF) or the National Health Card (CNS). Data record deterministic linkage can also be based on attributes inherent to each record, such as name, mother's name and date of birth, through the construction of a Statistical Linkage Key (SLK) by concatenating multiple variables^4^. On the other hand, the probabilistic linkage methods use statistical techniques to calculate the likelihood that two records refer to the same individual by comparing variables such as names, dates of birth, and other personal information. This approach enables data integration based on a defined level of similarity between the records^5^.

Many studies in Brazil have increasingly adopted RL methodologies to integrate databases. This growing trend highlights the importance of these techniques for health planning, surveillance, and epidemiological research. As a result, these processes are becoming more time-consuming and require computers equipped with high-performance hardware, including powerful processors and large random-access memory (RAM). One solution to address this challenge is the use of parallel or distributed computing, which optimizes processing time by distributing the workload across multiple cores or machines. Alternatively, another approach is to leverage tools specifically designed to handle large data sets efficiently without overloading machine memory or processing resources. Tools such as the DuckDB database management system offer in-process Structured Query Language (SQL) engines optimized for analytical workloads, allowing users to work with large volumes of data while minimizing computational overhead^6,7^.

The Mortality Information System (SIM) and the Influenza Epidemiological Surveillance Information System (SIVEP-Gripe) are two databases that contain information on patient deaths but do not have a unique identifier. This gap makes it difficult to identify the same individual in both databases, making it difficult to track and assess a person's complete health history. This study presents the accuracy of an algorithm for conducting SIM and SIVEP-Gripe RL tasks. Algorithms were developed for the RL process using deterministic and probabilistic methods within the DuckDB database environment, comparing their accuracy and computational feasibility with a Python-based algorithm previously validated in the literature^8,9^.

## METHODS

### Data sources and inclusion criteria

This study utilized data from SIM and SIVEP-Gripe. SIM, managed by the Brazilian Ministry of Health, records and monitors deaths nationwide, providing critical data for epidemiological surveillance and public health policies^10^. SIVEP-Gripe, in turn, tracks and analyzes cases of severe acute respiratory syndrome (SARS), playing a key role in monitoring outbreaks of respiratory diseases such as influenza and COVID-19^11^.

The SIM dataset (2015–2022) includes comprehensive mortality records, detailing causes of death, demographic characteristics, and clinical information. Meanwhile, the SIVEP-Gripe dataset (2020–2022) focuses on hospitalized severe acute respiratory illness (SARI) cases, particularly those related to COVID-19. Due to the urgency of data collection during pandemic peaks, the SIVEP-Gripe dataset presents unique challenges, such as data inconsistencies and reporting delays.

**Figure 1:**
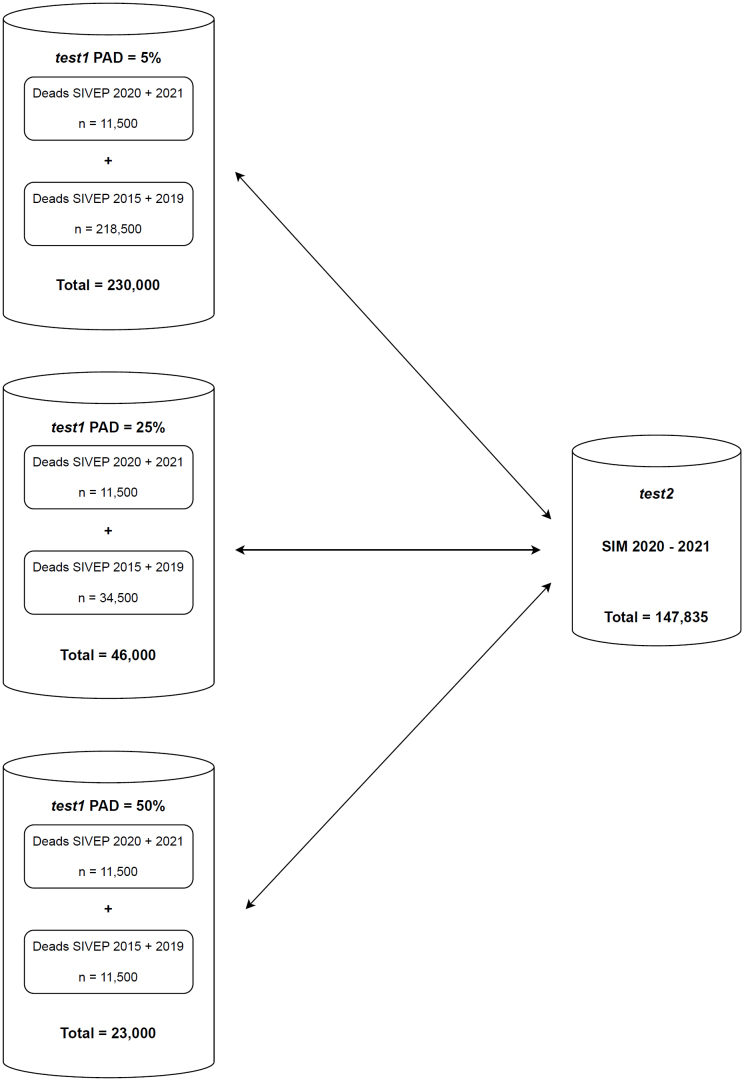
Flowchart of the Linkage Process in the 5, 25, and 50% Prevalence Scenarios for the *test1* Dataframe Compared to the *test2* Dataset.

To validate the RL process conducted in this study, two datasets were constructed using real-world data. The first dataset ("test1") consisted of a sample of true negative cases combined with a fixed set of 11,500 (24%) death records from SIVEP-Gripe for the years 2020–2021, which represented true positive cases. These positive cases were intentionally added to sampled records from the SIM database for the years 2015–2019, which represented records that should not be found in the second dataset. These samples were drawn in varying amounts to reflect different prevalence scenarios of true matches (PAD). Specifically, to simulate PAD levels of 10, 25, and 50%, we sampled 218,500, 34,500, and 11,500 SIM records, respectively. The second dataset ("test2") included all death records from the SIM database for the years 2020–2021. The objective was to assess whether the linkage method could accurately identify the true positives from "test1" within "test2" while minimizing false matches with true negatives (Figure 1).

PAD: people actually dead.

The strategies and parameters for the RL were defined based on a training sample from the two databases initially generated (Figure 1). In both algorithms, the RL was performed using the name, mother's name, and date of birth, tested across the three described scenarios. The performance of each method was evaluated based on accuracy parameters and execution time (measured in seconds), to assess both feasibility and computational efficiency.

### Record linkage algorithms and data preprocessing

The new linkage strategy presented employs a hierarchical approach that combines both deterministic and probabilistic methods to match records across databases. In the initial two steps, deterministic approaches are applied, relying on strict criteria, which requires exact matches for key variables such as name, mother's name, and date of birth.

Table 1 illustrates all the linkage strategies used, based on a hierarchical approach with stepwise refinement of matching criteria. The early steps use more stringent conditions, such as high similarity thresholds, along with specific blocking variables like first name or date of birth. As the process progresses, the criteria become less restrictive, enabling the algorithm to consider records with slight discrepancies in names or birth details. This sequential strategy ensures that records with the highest likelihood of accurate matches are prioritized and linked first, while those with lower similarity thresholds are assessed later in the process. This approach enhances both the precision and efficiency of the RL, as it systematically refines matches from the most to the least confident, reducing the potential for errors and optimizing overall accuracy.

**Table 1:** Strategies for record linkage: deterministic and probabilistic approaches.

Steps	Methods	Relationship Variables	Blocking Variables
1	Deterministic	SLK [name + mother's name + date of birth]	-
2	Deterministic	SLK [name + mother's name without the last surname + date of birth]	-
3	Probabilistic	jaro_winkler_similarity [name + mother's name + date of birth]/3>0.96	[first name, second name, first mother's name, second mother's name]
4	Probabilistic	jaro_winkler_similarity [name + mother's name]/2>0.95	[first name, second name, first mother's name, day of birth, month of birth]
5	Probabilistic	jaro_similarity [2 x name + mother's name]/3>0.90	[first name, second name, first mother's name, second mother's name, day of birth, month of birth]
6	Probabilistic	jaro_winkler_similarity [2 x name + mother's name]/3>0.91	[first name, second name, day of birth, month of birth]
7	Probabilistic	jaro_winkler_similarity [2 x name + mother's name]/3>0.96	[first name, day of date of birth, month of birth]
8	Probabilistic	jaro_winkler_similarity [name + mother's name without the last surname]/2>0.97	[first name, day of birth, year of birth]
9	Probabilistic	jaro_winkler_similarity [name without the last surname + date of birth]/2>0.99	[first name, second name, month of birth, year of birth]
10	Probabilistic	jaro_similarity [name + mother's name]/2>0.91	[first name, day of birth, year of birth]
11	Probabilistic	jaro_similarity [first name + second name + last name + first mother's name, second mother's name]/5>0.97	[day of birth, month of birth, year of birth]
12	Probabilistic	jaro_winkler_similarity [name + mother's name]/2>0.98	[date of birth, first mother's name]
13	Probabilistic	jaro_winkler_similarity [name]>0.95	[first mother's name, day of birth, month of birth, year of birth]

A SLK is a method used to generate a unique pseudo-identifier for individuals by combining selected variables that remain relatively stable over time. This approach is particularly useful in the absence of a common unique identifier, enhancing the accuracy of record matching across different datasets while preserving privacy. According to Karmel^12^, SLKs provide a reliable way to link records when deterministic linkage using primary keys is not possible, especially in large and complex databases where identifiers may be missing or inconsistent.

If no match is found at this stage, the algorithm transitions to probabilistic methods, leveraging similarity scores like Jaro and Jaro-Winkler metrics. These metrics facilitate the identification of potential matches even when minor discrepancies or typographical errors exist in the records, with predetermined thresholds indicating the confidence level needed for a match.

The Jaro metric is a string matching algorithm that measures the similarity between two strings by evaluating the number of matching characters and their transpositions^13^. The Jaro-Winkler metric enhances this by introducing a prefix scaling factor, giving greater weight to strings that share common prefixes. This adjustment makes the method particularly effective for matching names and short strings where prefixes hold significant importance, increasing the accuracy of linkage in cases with slight spelling variations^14,15^.

The proposed algorithm in this study is compared to the Python-based algorithm described in Pacheco et al.^8^. This reference algorithm is a hierarchical deterministic method developed to recover the vital status of human immunodeficiency virus (HIV) patients lost to follow-up by linking cohort databases to a mortality database in Rio de Janeiro. It integrates exact comparisons with controlled approximations, allowing minor errors in names and dates of birth, and employs a phonetic code adapted from Soundex to account for orthographic variations. Achieving a sensitivity of up to 96.5% and specificity of 100%, the method significantly reduced loss to follow-up and improved death detection, including cases not explicitly recorded as HIV/AIDS-related.

### Accuracy measures and statistical analysis

Sensitivity, specificity, negative predictive value (NPV), and positive predictive value (PPV) were calculated, considering death as the positive outcome. Confusion matrices were constructed by cross-referencing the true status of the patients (dead or alive) with the results of the linkage procedure (matched or not matched). The accuracy differences between the algorithms were evaluated using a paired study design, where both algorithms were applied to the same set of records for direct comparison^16^. For the diagnostic test analyses, 95% confidence intervals were used to ensure statistical reliability.

All RL procedures were conducted on a Linux Mint 21.3 Cinnamon system with a 5.15.0-119-generic x86_64 Linux kernel. The machine was equipped with an Intel Core i9-10900X CPU @ 3.7GHz 10 cores (20 threads), 64 GB of RAM, and an NVIDIA TU104GL [Quadro RTX 5000] graphics card. One of the linkage processes was performed using DuckDB version 0.10.0 (https://duckdb.org/) with the DBeaver interface version 24.2.0.2024 (https://dbeaver.io/), while the other process was conducted using Python version 3.10.12 (https://www.python.org/). Statistical analyses were conducted in the R software environment version 4.3.1 (https://cran.r-project.org/).

#### Data Availability Statement:

 The data used in this study include sensitive personally identifiable information, such as the individuals’ names and mother's names, extracted from original records in official health information systems. Due to the sensitivity of these data and in compliance with personal data protection regulations, the complete set cannot be made publicly available. Access may only be obtained upon formal request to the Rio de Janeiro Municipal Health Department, the authority responsible for storing and authorizing the use of the data.

## RESULTS

Table 2 presents the accuracy measures and processing times for progressive PAD. The proposed algorithm showed high accuracy indicators, with sensitivity in low prevalence scenarios equal to 94.2% [93.7; 94.6]. Specificity was measured punctually in 99,9 [99.9; 100.0].

**Table 2:** Accuracy criteria (95% confidence interval) for proposed and Pacheco et al.'s^8^ algorithms: complete data across various true positive prevalence scenarios.

Accuracy Criteria		Proposed algorithm	PACHECO et al.'s (2008) algorithm
5% PAD			
	Sensitivity	0.942 [0.937; 0.946]	0.927 [0.922; 0.932]
	TP/FN count	10,830 / 670	10,627 / 837
	PPV	0.998 [0.996; 0.998]	0.994 [0.992; 0.995]
	Specificity	0.999 [0.999; 1.000]	0.999 [0.999; 1.000]
	TN/FP count	218,473 / 27	218,425 / 66
	NPV	0.997 [0.997; 0.997]	0.996 [0.996; 0.996]
	Accuracy	0.997 [0.996; 0.997]	0.996 [0.996; 0.996]
25% PAD*			
	Sensitivity	0.940 [0.936; 0.945]	0.928 [0.924; 0.933]
	TP/FN count	10,814 / 686	10.649 / 851
	PPV	1.000 [0.999; 1.000]	0.999 [0.998; 1.000]
	Specificity	0.999 [0.999; 1.000]	0.999 [0.999; 1.000]
	TN/FP count	34,497 / 3	34,491 / 9
	NPV	0.981 [0.979; 0.982]	0.977 [0.975; 0.978]
	Accuracy	0.985 [0.984; 0.986]	0.982 [0.981; 0.983]
50% PAD*			
	Sensitivity	0.941 [0.934; 0.947]	0.926 [0.921; 0.930]
	TP/FN count	10,777 / 723	10,644 / 856
	PPV	1.000 [0.999; 1.000]	1.000 [0.999; 1.000]
	Specificity	1.000 [0.999; 1.000]	1.000 [0.999; 1.000]
	TN/FP count	11,500 / 0	11,498 / 2
	NPV	0.941 [0.937; 0.945]	0.931 [0.926; 0.935]
	Accuracy	0.969 [0.966; 0.971]	0.963 [0.960; 0.965]

PAD: people actually dead; PPV: positive predictive value; NPV: negative predictive value; TP/FN: true positives/false negatives; TN/FP: true negatives; false positives.

The proposed algorithm consistently exhibited slightly higher sensitivity compared to Pacheco's algorithm in the three tested prevalence scenarios (PAD). For 5% PAD, the proposed algorithm achieved 94.2% sensitivity, slightly surpassing Pacheco's algorithm with 92.7% of sensitivity. At 25% PAD, the proposed algorithm maintained a small advantage with 94.0% *versus* 92.8% for Pacheco's algorithm. Similarly, for 50% PAD, the proposed algorithm scored 94.1% compared to Pacheco's algorithm 92.6%. Although these differences are minor, they suggest that the proposed algorithm had a slight edge in correctly identifying true positives compared to Pacheco's algorithm. Both algorithms demonstrated a high specificity, nearing 100,0%. This means that both algorithms are highly accurate in identifying true negatives, minimizing the number of false positives.

Given the high sensitivity and specificity values, the NPV and PPV were markedly high, as expected. The proposed algorithm showed a slight edge in NPV, particularly in lower PAD scenarios. At 5% PAD, the proposed algorithm's NPV was 99.7%, marginally outperforming 99.6% for Pacheco's algorithm. For 25% PAD, the proposed algorithm also maintained a small advantage with 98.1% compared to 97.7% for Pacheco's algorithm. At 50% PAD, the NPV was also slightly higher than Pacheco's algorithm. These results suggest that the proposed algorithm has a marginally better ability to correctly identify true negatives (Table 2).

The most valuable result of the proposed algorithm focuses on processing speed (Table 3). The proposed algorithm achieved considerable gains in linkage processing speed, in all evaluated contexts. The processing speed was greater as the prevalence decreased.

**Table 3:** Processing time (seconds) for proposed and Pacheco et al.'s^8^ algorithms: complete data across progressive true positive prevalence scenarios.

Prevalence PAD (%)	Processing Time (seconds)	
	Proposed Algorithm	Pacheco et al.'s (2008) algorithm
5	38.48	4,566.82
25	19.52	966.2
50	15.52	515.35

PAD: people actually dead.

For 5% PAD, the proposed algorithm took just 38.48 seconds, vastly outperforming 4,566.82 seconds for Pacheco's algorithm. For 50% PAD, the proposed algorithm processed the data in 15.52 seconds, while Pacheco's algorithm required 515.35 seconds. The difference in processing time underscores the proposed algorithm's efficiency, especially in handling large datasets or more complex queries, making it a highly effective choice especially in low-prevalence scenarios.

## DISCUSSION

The overall accuracy of both algorithms remained stable across different prevalence scenarios. However, the proposed algorithm demonstrated a slight advantage in sensitivity while maintaining high specificity, similar to Pacheco's algorithm. The most notable difference was its processing speed; its implementation in DuckDB executed the same procedure in significantly less time. This makes the DuckDB-based algorithm more efficient for tasks requiring fast execution, particularly in scenarios involving large datasets and real-time processing needs. Moreover, it maintains its efficiency even when handling high data volumes, making it a robust solution for complex applications in public health.

Beyond its computational efficiency, the proposed algorithm proves to be well-suited for large-scale RL tasks. The results suggest that DuckDB is a promising tool for public health applications, where integrating extensive datasets in a timely manner is essential. Notably, as the prevalence of true positive records increased, DuckDB's processing time decreased while preserving high accuracy, reinforcing its effectiveness. This combination of speed, precision, and scalability makes it a highly practical choice for time-sensitive data linkage tasks, demonstrating its potential for handling large-scale public health datasets with both accuracy and speed.

The SIM and the SIVEP-Gripe are two crucial databases for public health surveillance and epidemiological monitoring in Brazil. These databases play a fundamental role in tracking severe respiratory infections, assessing disease burden, and informing public health interventions. During the COVID-19 pandemic, both systems were extensively used, either independently or in studies that linked their records using deterministic and/or probabilistic approaches^17^. The integration of these datasets through RL allows for a more comprehensive understanding of disease progression, fatality rates, and patient outcomes by connecting hospitalization data (SIVEP-Gripe) with mortality records (SIM). This enhances the completeness and consistency of information, supporting more accurate epidemiological analyses and better-informed decision-making in public health.

The proposed algorithm for RL, in the Brazilian context, is highly adaptable, making it suitable for various administrative levels—from resource-constrained municipal health departments to the federal Ministry of Health. Its efficiency allows for the integration of large datasets at the national scale without requiring expensive or advanced hardware infrastructure, ensuring accessibility even in settings with limited computational resources.

The relationship between databases through RL is a powerful strategy in public health, as it enhances data completeness, enables longitudinal follow-up, and strengthens decision-making processes. By integrating multiple data sources, linkage allows researchers and policymakers to construct more comprehensive individual and population health profiles, facilitating epidemiological surveillance, disease monitoring, and resource allocation. This method improves the detection of repeated records, reducing redundancy and inconsistencies while enhancing data accuracy. Additionally, RL supports retrospective studies and predictive modeling by consolidating fragmented information, ultimately improving healthcare planning and intervention strategies.

On the other hand, the limitations of a data RL algorithm include several challenges that impact its accuracy and efficiency. Incomplete or missing data, such as absent name fields, dates of birth, or addresses, complicates the matching process, while typing errors and inconsistencies, such as misspellings, make it difficult to identify whether two records refer to the same entity. Homonyms and false positives, where individuals or entities with identical names are incorrectly linked, further reduce reliability, as do format variations in data such as dates, addresses, or names. The scalability of data is another significant challenge, as the number of comparisons increases exponentially with the dataset size, necessitating medium-to-high processing power to ensure satisfactory performance. Additionally, the choice of similarity metrics, such as Levenshtein or Jaro-Winkler, can greatly influence the algorithm's effectiveness, and assessing the quality of RL is challenging without a ground truth dataset for validation. Furthermore, this specific algorithm requires familiarity with SQL, limiting its accessibility to users without prior expertise in the language. These challenges underscore the complexity of implementing and operating RL algorithms effectively.

In the municipality of Rio de Janeiro, Brazil, the RL methods employed here have been instrumental in constructing and updating the CARIOCA database^18^. Stored in the city's centralized data lake, the CARIOCA database is a retrospective digital cohort comprising approximately 7 million records of individuals vaccinated and/or affected by COVID-19 within the municipality. The database will be incrementally updated through the upload of datasets initially used in its creation. These include the SIM, SIVEP-Gripe, e-SUS, and the National Immunization Program Information System SI-(PNI). Additionally, other health information systems, such as the Disease Notification System (SINAN), Live Birth Information System (SINASC), and Hospital Information System (SIH), will be incorporated into the database.

Integration of health information systems is an important strategy described in the Brazilian National Health Surveillance Policy^19^. The continuous and growing access to the volume of records in these systems, along with the need to enhance linkage processes in terms of both accuracy and speed, has driven the development of new linkage strategies to fully leverage the information generated. These advancements aim to optimize data integration, reduce redundancy, and improve the quality of epidemiological analyses, reinforcing the role of RL as a critical tool for public health surveillance and decision-making.

This comparison highlights the innovation of the proposed algorithm, which introduces a novel technology compared to an established used method for this type of analysis. By contrasting the new approach with a traditional technique, the goal is to showcase the potential enhancements and advancements that the new algorithm and technology can bring to the RL process.

The comparison between the two algorithms showed that the performance of the algorithm discussed in this article is comparable to that of Pacheco's Python-based algorithm, while offering unique advantages. The main innovations include its ability to run efficiently on modest computing resources—even on low- to mid-range machines—leveraging SQL, a well-established and intuitive language, along with a significant boost in processing speed. These qualities make the proposed method not only highly effective but also capable of matching the efficiency of the traditional approach, with added benefits that enhance the RL process, all while utilizing free and open-source software.
